# Errors in Byline

**DOI:** 10.1001/jamanetworkopen.2024.42253

**Published:** 2024-10-04

In the Research Letter titled “Using AI and Social Media to Understand Health Disparities for Transgender Cancer Care,”^1^ published August 23, 2024, the name and academic degrees of Al Asante-Facey, PA-C, MBA, were listed incorrectly in the byline. This article has been corrected.^1^

## References

[1] Annan A, Li Y, Du J, . Using AI and social media to understand health disparities for transgender cancer care. JAMA Netw Open. 2024;7(8):e2429792. doi:10.1001/jamanetworkopen.2024.29792 39178002 PMC11344235

